# Preparation of Polyimide/Graphene Oxide Nanocomposite and Its Application to Nonvolatile Resistive Memory Device

**DOI:** 10.3390/polym10080901

**Published:** 2018-08-11

**Authors:** Ju-Young Choi, Hwan-Chul Yu, Jeongjun Lee, Jihyun Jeon, Jaehyuk Im, Junhwan Jang, Seung-Won Jin, Kyoung-Kook Kim, Soohaeng Cho, Chan-Moon Chung

**Affiliations:** 1 Department of Chemistry, Yonsei University, Wonju, Gangwon-do 26493, Korea; cjy0510@yonsei.ac.kr (J.-Y.C.); taken4@nate.com (H.-C.Y.); jinsw0906@yonsei.ac.kr (S.-W.J.); 2 Department of Physics, Yonsei University, Wonju, Gangwon-do 26493, Korea; leejj0858@yonsei.ac.kr (J.L.); jihyunn1211@yonsei.ac.kr (J.J.); jaehyuk5457@yonsei.ac.kr (J.I.); jgh6758@yonsei.ac.kr (J.J.); 3 Department of Nano-Optical Engineering, Korea Polytechnic University, Siheung 15073, Korea; kim.kk@kpu.ac.kr

**Keywords:** polyimide nanocomposite, graphene oxide, nonvolatile resistive memory, RRAM, WORM

## Abstract

2,6-Diaminoanthracene (AnDA)-functionalized graphene oxide (GO) (AnDA-GO) was prepared and used to synthesize a graphene oxide-based polyimide (PI-GO) by the in-situ polymerization method. A PI-GO nanocomposite thin film was prepared and characterized by infrared (IR) spectroscopy, thermogravimetric analysis (TGA) and UV-visible spectroscopy. The PI-GO film was used as a memory layer in the fabrication of a resistive random access memory (RRAM) device with aluminum (Al) top and indium tin oxide (ITO) bottom electrodes. The device showed write-once-read-many-times (WORM) characteristics with a high ON/OFF current ratio (I_on_/I_off_ = 3.41 × 10^8^). This excellent current ratio was attributed to the high charge trapping ability of GO. In addition, the device had good endurance until the 100th cycle. These results suggest that PI-GO is an attractive candidate for applications in next generation nonvolatile memory.

## 1. Introduction

Graphene, a two-dimensional honeycomb lattice substance, has attracted significant attention due to its’ exceptional mechanical, thermal, electronic, electrochemical and optical properties [1,2,3,4,5]. Graphene shows an excellent electron charge mobility of about 200,000 cm^2^/V at room temperature, and a low resistivity of about ~10^−6^ Ω cm [6]. However, it is difficult to incorporate pristine graphene sheets into polymer matrices due to its strong interlayer cohesive energy and surface inertia [7,8]. To overcome these limitations, graphene is converted to graphene oxide (GO) by chemical methods [9,10,11]. It is well known that good dispersion of nanoparticles and strong interfacial interaction between nanoparticles and polymers are two important factors that enhance the physicochemical properties of nanocomposites [12,13]. In order to further increase the dispersibility of GO, chemical functionalization utilizing the active sites on GO have been carried out successfully [14,15]. For example, several graphene derivatives modified with amine through nucleophilic substitution reaction [16,17], amidation reaction [18,19], phase transfer protocol [20] etc., have been reported. In-situ polymerization has also been demonstrated as an effective way [13,21,22,23,24] to avoid the aggregation of GO and to improve the interaction between GO and other components. GO has attracted significant interest because it can easily be functionalized and applied to various fields. In particular, GO has been demonstrated to be a promising candidate for dielectric materials [25] or charge trapping layers [26,27].

Polyimides (PIs) are now widely used for a variety of applications, including its use in display, aerospace, vehicles, and electronic industries due to its good mechanical properties, flexibility, excellent thermal stability, low dielectric permittivity and chemical resistance [28,29,30,31]. Recently, versatile PIs have been developed for application to high-temperature resistive random access memory (RRAM) [32]. RRAM has various advantages including fast speed, low power consumption and high storage density, and is considered to be one of the potential candidates for next generation nonvolatile memory [33,34,35]. PI-based RRAM is one of the important candidates due to good thermal stability and chemical resistance [36,37,38]. There are three RRAM types based on operation: bipolar, unipolar, and Write-Once-Read-Many-Times (WORM) RRAMs. Bipolar RRAM can be turned ON and OFF many times by applying opposite voltage polarities [33] while unipolar RRAM by two different voltages (low and high) with the same polarity [37]. Then, WORM RRAM is turned ON only once and keeps its status permanently [38] like a writable compact disc.

To the best of our knowledge, reports regarding RRAM derived from GO-based PI (PI-GO) nanocomposite are very rare. Recently, Wu et al. investigated the rewritable memory effect of a resistive switching device having Ag/PI/PI-GO/PI/indium-tin-oxide (ITO) structure [27]. In their work, a PI derived from 3,3′,4,4′-biphenyltetracarboxylic dianhydride (BPDA) and *p*-phenylenediamine (PDA) was employed as a matrix layer due to its insolubility in organic solvents and thermal stability. However, the fabrication of multi-layer memory device is complicated because of the repeated spin-coating processes, which may be troublesome and cause instability of the memory behavior. In the present study, we developed a simpler-structure memory device with a single PI-GO memory layer for application to RRAM device. Furthermore, the memory layer in this work is composed of GO and a PI derived from rel-(1′R,3S,5′S)-spiro[furan-3(2H),6′-[3]oxabicyclo[3.2.1]octane]-2,2′,4′,5(4H)-tetrone (DAn) and 2,6-diaminoanthracene (AnDA). The anthracene-containing PI film was reported to show resistive memory behavior and optical transparency [38]. The PI-GO nanocomposite film was prepared and used to fabricate an ITO/PI-GO/aluminum (Al) memory device and its memory behavior was investigated. An ITO/organic layer/Al structure is widely used for organic RRAM devices [39].

## 2. Materials and Methods

### 2.1. Materials

DAn was donated by Prof. K. Kudo (Tokyo University). AnDA was prepared by using a previously reported method [40]. GO sheets were purchased from Dae Joo Electronic Materials Co., Ltd., Gyeonggi-do, Korea, and used without purification. *N*,*N*-dimethylacetamide (DMAc)), acetic anhydride and pyridine were purchased from Duksan Pure Chemicals Co., Ltd., Seoul, Korea. The rest of the chemicals were of analytical grade and used without further purification. 

### 2.2. Characterization

Infrared (IR) spectra were acquired using a PerkinElmer Spectrum One B FT-IR spectrometer, in the range of 500 to 4000 cm^−1^ at a resolution of 4 cm^−1^ with KBr pellet technique. UV-visible spectra were recorded on a Perkin Elmer Lambda 25 UV/Vis spectrometer. Thermal analyses were carried out under the nitrogen atmosphere with a flow rate of 50 mL/min using a Shimadzu TGA-50 instrument at a heating rate of 10 °C/min. The field emission scanning electron microscope (FE-SEM) used in this work was a Hitachi SU-70 (Hitachi Ltd., Tokyo, Japan), and measurements were conducted using an acceleration voltage of 20 or 30 kV and a working distance of 10 mm. Spin-coating was performed using an ACE-200 spin coater (Dong Ah Trade Corp, Seoul, Korea). Current-voltage (I-V) characteristics were measured using a Keithley 2634 sourcemeter under ambient air and temperature conditions. The voltage was applied to the top electrode while the bottom electrode was grounded, and measurements were conducted utilizing a tungsten tip.

### 2.3. Preparation of PI-GO

GO (0.1362 g) was homogeneously dispersed in 20 mL of dimethylacetamide (DMAc) by tip sonication for 1 h. The dispersed GO suspension was poured into a 100-mL three-necked flask equipped with a magnetic stirrer and under the nitrogen atmosphere. AnDA (85.4 mg, 0.41 mmol) was added and the resultant mixture was stirred at 60 °C for 24 h to give a AnDA-modified GO (AnDA-GO). Then, DAn (2.24 g, 0.01 mol) and AnDA (2.08 g, 0.01 mol) were put into the flask. The mixture was stirred at the room temperature for 24 h and then chemical imidization was conducted by the addition of acetic anhydride and pyridine and subsequent heating at 160 °C for 6 h in a reflux system [41]. After the reaction, the PI-GO concentration was controlled to 1 wt % by adding DMAc to prepare a PI-GO solution for spin-coating.

### 2.4. Preparation of PI-GO Memory Device

The PI-GO solution was spin-coated onto a cleaned ITO glass substrate (ITO thickness = 125 nm). The resultant film was heated at 60 °C for 24 h to completely remove the solvent (PI-GO film thickness = 65 nm). Circular Al top electrodes were prepared by thermal evaporation under 10^−7^ Torr via a shadow mask with a diameter of 200 μm.

## 3. Results and Discussion

### 3.1. Preparation of PI-GO

**Figure 1:**
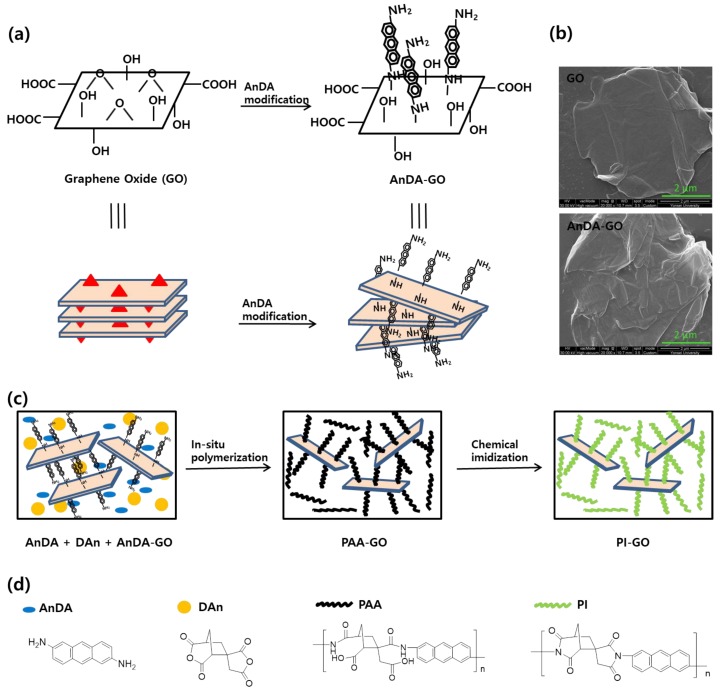
Schematic illustrations of the synthesis of (**a**) AnDA-GO and (**c**) PI-GO; (**b**) FE-SEM images of the GO and AnDA-GO; (**d**) Chemical structures of AnDA, DAn, PAA and PI.

PI-GO was prepared through an in situ polymerization process [42]. After the GO powder was dispersed in DMAc using tip sonication, AnDA was added to modify the GO. The GO modified with AnDA is represented as AnDA-GO (Figure 1a). Oxygen functionalities of GO would bind with H_2_O molecules, resulting in a smooth surface morphology (Figure 1b, top image) [42]. Compared to GO sheets, AnDA-GO shows a rough surface morphology (Figure 1b, bottom image), totally different from that of the as-prepared GO [43,44]. The AnDA monomer is thought to attach to the GO sheet surface through a nucleophilic attack by the amino group on the epoxy group [42,45,46,47]. A mixture of DAn, AnDA and AnDA-GO in DMAc was stirred at the room temperature for 24 h to prepare poly(amic acid)-GO (PAA-GO), which was then chemically imidized to obtain PI-GO (Figure 1c). The prepared PI-GO contained 3 wt % of GO. Figure 1d shows the chemical structures of the monomers and polymers used in this work.

### 3.2. Characterization of PI-GO

**Figure 2:**
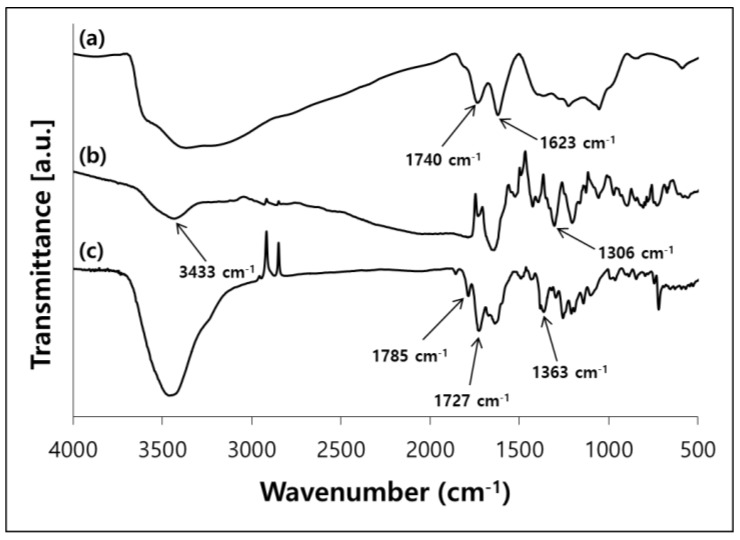
FT-IR spectra of (**a**) GO; (**b**) AnDA-GO and (**c**) PI-GO.

The chemical structures of GO, AnDA-GO and PI-GO were confirmed through their FT-IR spectra (Figure 2). The IR spectrum of GO exhibited two characteristic bands at 1740 and 1623 cm^−1^, which corresponded to C=O (carboxyl) and C=C skeleton stretching vibrations, respectively (Figure 2a) [46,48,49]. AnDA-GO showed absorption bands at 1306 cm^−1^ due to C–N stretching, and at 3433 cm^−1^ due to secondary N–H stretching (Figure 2b) [49,50]. PI-GO showed characteristic imide absorption bands at 1785 cm^−1^ due to imide C=O asymmetric stretching, at 1727 cm^−1^ due to imide C=O symmetric stretching, and at 1363 cm^−1^ due to imide C–N stretching, suggesting the formation of PI (Figure 2c) [51,52,53].

**Figure 3:**
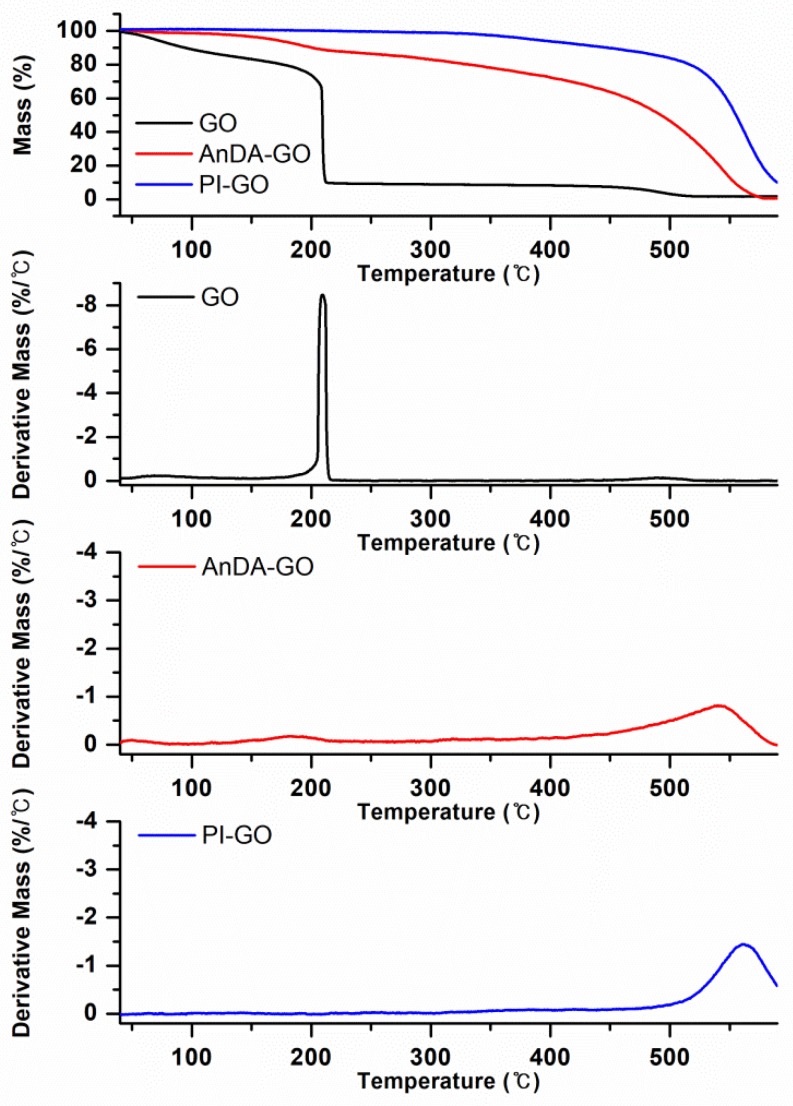
TGA and DTG thermograms of GO, AnDA-GO and PI-GO.

Thermal decomposition of the samples was studied by thermogravimetric analysis (TGA) under the nitrogen atmosphere at a heating rate of 10 °C/min. The data are presented in Figure 3, showing TGA and differential thermogravimetry (DTG) thermograms of GO, AnDA-GO and PI-GO. The TGA curve of GO showed three major weight losses [49,52,54]. The first weight loss occurred around 72 °C due to the loss of residual H_2_O contained in GO, while the second weight loss occurred around 209 °C due to rapid expansion [55,56,57] and the decomposition of the labile oxygen-containing functional groups such as epoxy, hydroxyl, carboxyl [45,46,49,58]. The last weight loss around 480 °C was ascribed to the removal of more stable oxygen functionalities [54,59]. Compared to the decomposition behavior of GO, AnDA-GO exhibited a small weight loss curve due to the replacement of oxygen groups by amine groups. PI-GO showed the high thermal stability of 10% weight loss (*T*_10_) at 449 °C. The stable thermal properties of PI-GO is attributed to the build-up of strong inter- and/or intra-molecular interactions [15,60] and the thermal stability of the imide ring of PI [61].

**Figure 4:**
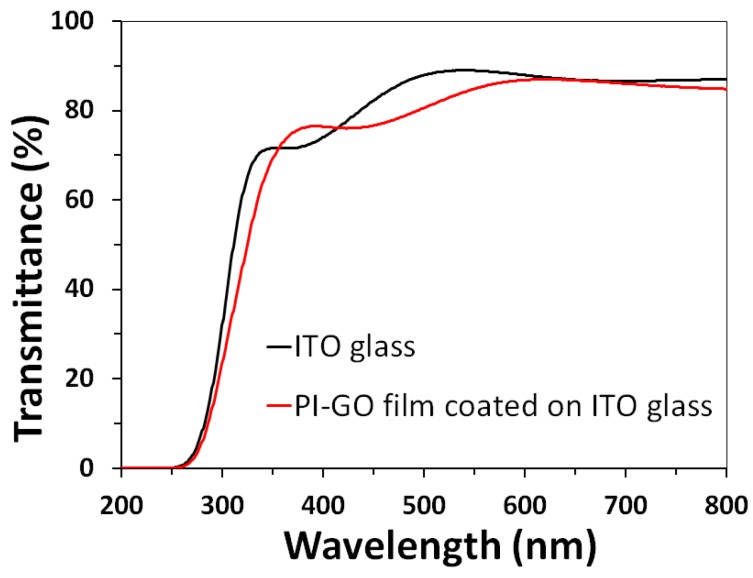
UV-vis spectra of a ITO glass and a PI-GO film coated on ITO glass.

Figure 4 shows UV-visible spectrum of a PI-GO film coated on ITO glass (film thickness = 65 nm). The transmittance of an ITO glass substrate was also measured for comparison. The two spectra showed the similar transmittance of about 75% at 400 nm and about 85% at 550 nm. It is evident that the PI-GO film has high optical transparency similar to that of the ITO glass in the visible region. The high transparency of the PI-GO film can be attributed to the low degree of conjugation and charge transfer complex resulting from the aliphaticity and unsymmetrical spiro structure of the DAn unit [38]. The high transparency of the PI-GO film can lead to the potential development of transparent memory device if the transparent top and bottom electrodes are used.

### 3.3. Fabrication of PI-GO-Based Memory Device

**Figure 5:**
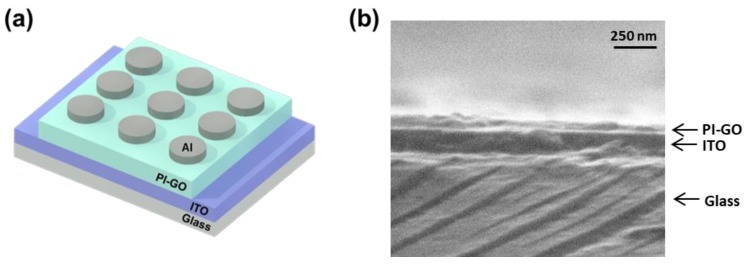
(**a**) Schematic structure of the ITO/PI-GO/Al device; (**b**) FE-SEM image of the PI-GO on ITO glass.

We fabricated a memory device using PI-GO as an active layer (Figure 5). The PI-GO solution was spin-coated onto a cleaned ITO glass substrate, and the resultant film was heated at 60 °C to completely remove the solvent (film thickness = 65 nm) (Figure 5b). Circular Al top electrodes were prepared by thermal evaporation via a shadow mask with a diameter of 200 μm (Figure 5a).

### 3.4. Memory Device Characteristics

**Figure 6:**
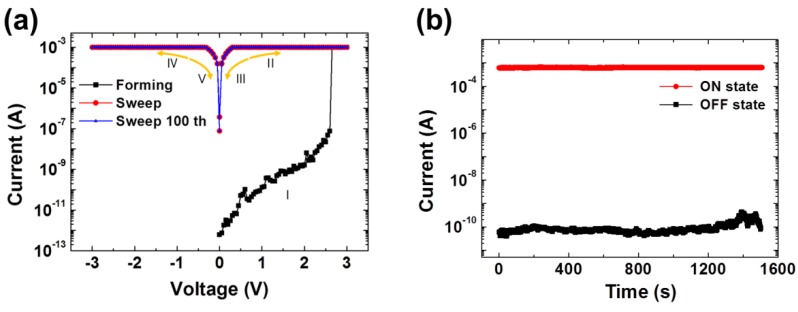
(**a**) I-V characteristic of the ITO/PI-GO/Al device under ambient air conditions at room temperature; (**b**) Retention performance of the ITO/PI-GO/Al device at room temperature (Read at 0.2 V).

Figure 6a shows representative I-V characteristic curves of the PI-GO memory device with a compliance current of 1 × 10^−3^ A. Initially, this device showed a gradual increase in the current until an applied voltage of 2.65 V. Afterwards, the current rapidly increased over the voltage of 2.65 V. This transition indicates the forming process (from OFF state to ON state) of the device and it can play a role in the “writing” process of the digital memory device [38]. Besides, during the sweep of the voltage from 0 to 3.5 V (positive region) and from 0 to −3.5 V (negative region), no specific degradation was observed, therefore, the device can be classified as a WORM memory device [38]. In particular, the device showed an ON/OFF current ratio of 3.41 × 10^8^, which is excellent compared to the reported current ratios of polymer-based memory devices with WORM memory characteristics [32]. The good ON/OFF current ratio of ITO/PI-GO/Al device is attributable to the high charge trapping ability of GO [26,27]. The ON state current was steadily maintained until the 100th sweep; in other words, the endurance of the device is acceptable [62,63].

Unlike bipolar and unipolar RRAMs which go through multiple ON and OFF processes, our device is a WORM memory. Therefore, once the device is turned ON, it is permanently ON, which is exactly the same as a writing (data recording) process of the writable compact disc. Also, we performed the sweep measurement with as-grown samples in a probe station open to ambient air without protecting any post-process (protection from humidity or oxidation). The ON state was still clearly well maintained after a 100-cycle sweep as shown in Figure 6a. Figure 6b shows the retention ability of the device at 0.2 V at ambient air and room temperature conditions. The device maintained both the ON and OFF state for 1500 s without any degradation and this stability as a WORM memory device is suitable for a digital memory device [37,64]. The post-process for protecting device and packaging could further improve reliability in practice.

**Figure 7:**
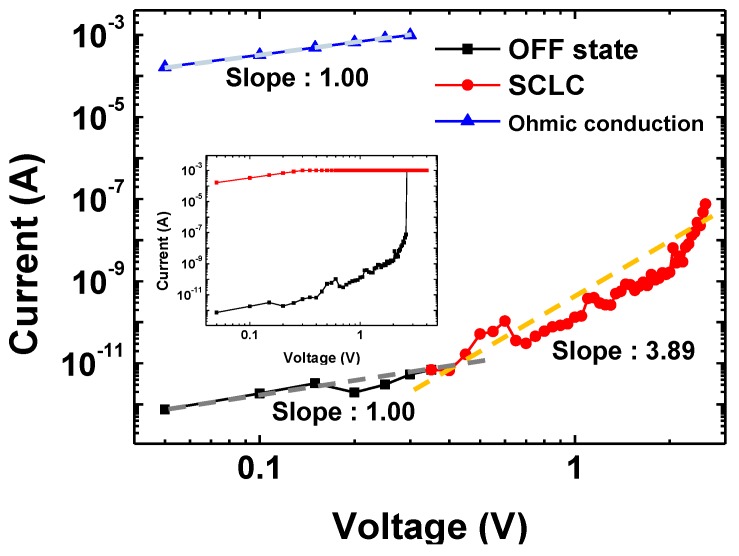
Experimental data and fitted lines of the I-V characteristics in the OFF state and ON state (Inset: original I-V characteristic of the ITO/PI-GO/Al device).

Figure 7 shows the log I–log V plot of the original I-V characteristic of the ITO/PI-GO/Al device’s positive region (Figure 7, inset). The current shows two distinct regions. In the initial OFF state (black line), the slope maintains a linear current behavior (<0.4 V) with a slope of 1.00, which exhibits Ohmic conduction [65]. When increasing the voltage (>0.4 V), another behavior was observed with a slope of 3.89 (red line), which indicated the trap-limited space charge limited current (SCLC) state. In the SCLC conduction model, the current density (*J*) can be represented as follows [66]:

(1) $$J\;\propto d{\left(\frac{V}{d^{2}}\right)}^{m},$$

where *V*, *d* and *m* represent the applied voltage, the sample thickness and the fitting parameter, respectively. An insulator having a specific *m* value of 2 possesses no free carriers at equilibrium and no bulk traps. However, in the case of *m* values that range from 2 to 11, the device has many charge traps in the active layer [66]. In this voltage region (>0.4 V), a lot of charge carriers are inserted and trapped in the PI-GO layer. Therefore, these space charges could restrict the device current and govern resistive switching. Additionally, the device’s I-V characteristic of having a slope of 3.89 can be explained by a SCLC mechanism. Meanwhile, the ON state (blue line) revealed Ohmic conduction with a slope of 1.00, indicating a typical Ohmic contact region induced by thermally generated free charge carriers [65]. These results suggest that WORM memory property of the PI-GO film is controlled by the trap-controlled SCLC and Ohmic conduction model [35,66].

## 4. Conclusion

AnDA-GO was prepared and used to synthesize a PI-GO nanocomposite. ITO/PI-GO/Al RRAM device was fabricated with a single PI-GO nanocomposite memory layer. Its fabrication process was much simpler than the previously reported multi-layer process [27]. Our device revealed WORM characteristics with an excellent ON/OFF current ratio of 3.41 × 10^8^, endurance until the 100th cycle and retention during the 1500 s. This good ON/OFF current ratio was attributable to high charge trapping ability of GO. These results indicate that PI-GO is a promising material for application in the next generation non-volatile memories.
